# Book Reviews

**Published:** 1877-03-20

**Authors:** 


### BOOK REVIEWS.

A PRACTICAL TREATISE ON DISEASES OF THE SKIN, by Louis A. Duhring, M. D., Professor of Diseases of the Skin in the Hospital of the University of Pennsylvania, Physician to the Dispensary for Skin Diseases, Philadelphia. Author of Atlas of Skin Diseases, etc.

The above valuable work is just issued from the publishing house of J. Lippincott & Co., Philadelphia. The author remarks that in preparing the volume it was his "aim to write a concise, practical and useful treatise—one which, while making no pretensions to being exhaustive, should comprise sufficient to afford a clear insight into the elements of Dermatology, and a knowledge of all the important facts in connection with each disease treated of. The primary object being to render the subject simple and intelligible and to free it from unnecessary encumbrances, it has been deemed best to avoid all questions of theory, discussion of unsettled points, and the introduction of useless or obsolete terms."

In perusing the work we find that the author has well complied with the objects above expressed, and has given a truly concise and practical work wherein all that is valuable is presented in a manner which must prove highly interesting and acceptable to the practitioner. Having been a pupil of the renowned Ferdinand Huber, Professor of Dermatology in the University of Vienna, and having had unusually favorable opportunities for observation in the various countries of Europe, and also in the United States, Professor Duhring is eminently fitted for preparing a work on skin affections, and well and truly has he performed the task.

